# Investigation of the Side Chain Effect on Gas and Water Vapor Transport Properties of Anthracene-Maleimide Based Polymers of Intrinsic Microporosity

**DOI:** 10.3390/polym14010119

**Published:** 2021-12-29

**Authors:** Esra Caliskan, Sergey Shishatskiy, Silvio Neumann, Volker Abetz, Volkan Filiz

**Affiliations:** 1 Institute of Membrane Research, Helmholtz-Zentrum Hereon, Max-Planck-Str. 1, 21502 Geesthacht, Germany; esra.caliskan@hereon.de (E.C.); sergey.shishatskiy@hereon.de (S.S.); silvio.neumann@hereon.de (S.N.); volker.abetz@hereon.de (V.A.); 2 Institute of Physical Chemistry, University of Hamburg, Martin-Luther-King-Platz 6, 20146 Hamburg, Germany

**Keywords:** polymers of intrinsic microporosity (PIM-1), membranes, gas separation, water vapor transport, Diels Alder reaction

## Abstract

In the present work, a set of anthracene maleimide monomers with different aliphatic side groups obtained by Diels Alder reactions were used as precursors for a series of polymers of intrinsic microporosity (PIM) based homo- and copolymers that were successfully synthesized and characterized. Polymers with different sizes and shapes of aliphatic side groups were characterized by size-exclusion chromatography (SEC), (nuclear magnetic resonance) ^1^H-NMR, thermogravimetric (TG) analysis coupled with Fourier-Transform-Infrared (FTIR) spectroscopy (TG-FTIR) and density measurements. The TG-FTIR measurement of the monomer-containing methyl side group revealed that the maleimide group decomposes prior to the anthracene backbone. Thermal treatment of homopolymer methyl-100 thick film was conducted to establish retro-Diels Alder rearrangement of the homopolymer. Gas and water vapor transport properties of homopolymers and copolymers were investigated by time-lag measurements. Homopolymers with bulky side groups (*i*-propyl-100 and *t*-butyl-100) experienced a strong impact of these side groups in fractional free volume (FFV) and penetrant permeability, compared to the homopolymers with linear alkyl side chains. The effect of anthracene maleimide derivatives with a variety of aliphatic side groups on water vapor transport is discussed. The maleimide moiety increased the water affinity of the homopolymers. Phenyl-100 exhibited a high water solubility, which is related to a higher amount of aromatic rings in the polymer. Copolymers (methyl-50 and *t*-butyl-50) showed higher CO_2_ and CH_4_ permeability compared to PIM-1. In summary, the introduction of bulky substituents increased free volume and permeability whilst the maleimide moiety enhanced the water vapor affinity of the polymers.

## 1. Introduction

Membrane technology has drawn great attention owing to its advantages of being ecologically favorable, cost-efficient and easy to operate. Polymers are encouraging materials for gas separation membranes due to their benign processability and low upscaling costs [1]. In gas separation, there are two significant parameters that define membrane performance, permeability (*P*) and selectivity (*α* = *P_A_*/*P_B_*). Permeability is a product of solubility (*S*) and diffusivity (*D*) (Equation (1)) according to the solution-diffusion model [2]. The selectivity parameter, therefore, is an indirect function of solubility and diffusivity of the membrane. The aforementioned transport parameters are directly affected by the state and microstructure of the membrane material; thus, it is crucial to develop particularly tailored polymers for gas separation application [3]. Usually, glassy polymers are used for the separation of non-condensable gases, and for these polymers, a trade-off between permeability and selectivity is known to cause limits to the use of polymers in practical gas separation [4]. In general, gas transport takes place through interconnected free volume elements formed by non-perfect packing of macromolecular chains of amorphous polymers. Consequently, polymer membranes have difficulties in offering both high permeability and high selectivity, which are mostly defined by large free volume size and narrow size distribution, respectively [5].

(1) P=DS

Researchers endeavor to overcome permeability and selectivity trade-offs by developing new polymeric materials based on the consideration of empirical structure/property interconnections. A breakthrough discovery addressing this issue was the development of polymers of intrinsic microporosity (PIM) by Budd and McKeown, which have a fractional free volume comparable to disubstituted polyacetylenes [6]. Among the polyacetylenes, poly(trimethylsilylpropyne) (PTMSP) is known to be the most permeable polymer so far. Nevertheless, PTMSP has selectivity drawbacks due to its insufficient rigid structure, which leads to failure of size discrimination [7,8]. Hence, PIMs have drawn attention during the past years owing to their unique properties. The term intrinsic microporosity refers to a network of interconnected free volume voids formed by highly inefficient packing of rigid and contorted macromolecules [9]. The essence of the existence of microporosity in a polymer lies in the presence of a contortion site in the monomeric unit, which could be obtained either by the introduction of a spiro- center, a non-planar rigid conformation or a single covalent bond that limits the rotation within the polymer chain [10]. Bearing these specificities in mind, researchers have focused on the development of different PIMs. PIM-1 is the most extensively studied polymer among the PIM family, which is obtained by the polycondensation of 5,5′,6,6′-tetrahydroxy-3,3,3′,3′-tetra-methylspirobisindane (TTSBI) and 2,3,5,6-tetrafluoroterephthalonitrile (TFTPN) [11]. Since its discovery, PIM-1 has become attractive due to its high solubility in low boiling point solvents such as tetrahydrofuran (THF) or chloroform (CHCl_3_), accessibility to chemical modifications, high surface area accompanied by attractive gas separation properties [10]. Owing to its splendid features, PIM-1 has been studied in many membrane applications such as gas separation [12,13,14,15], hydrogen storage [16], pervaporation [6] and water separation [17,18]. Although extensive research has been carried out on PIMs, a development of tailoring PIM for membrane applications, especially for gas separation, is still necessary.

The literature on intrinsic microporosity has highlighted several ways to ameliorate gas transport performance. Previous research focused on post-modification of PIMs to enhance selectivity by the introduction of CO_2_ affine functionalities such as amide [19], amine [20], carboxylic acid [21], hydroxyl [22], tetrazole [23], thioamide [24] and vinyl [25]. The cornerstone of these studies is based on the exploitation of CO_2_-philic groups by increasing CO_2_ solubility, thus upgrading the selectivity without compromising permeability. Further studies examined blend preparation with microporous fillers, e.g., carbon nanotubes [26], graphene oxide [27], MOF [28] and POSS [29]. One of the other aims of these studies was to tackle physical aging, a common problem with glassy polymers.

In addition to the abovementioned studies, a different approach employed by a vast number of researchers is to develop a shape-persistent polymer backbone by introducing rigid or non-coplanar monomers [30,31]. The incorporation of rigid groups into the polymer backbone disrupts the chain packing and consequently increases the free volume [32]. Considering this approach, Bezzu et al. [15] replaced the spirobisindane group with a relatively more rigid spirobifluorene and succeeded in ensuring good permeability-selectivity interrelation. The introduction of Tröger’s base and ethanoanthracene into PIM structures (PIM-SBI-TB [8,33] and PIM-EA-TB [33], respectively) enhanced the sieving property of the polymer owing to inflexible, rigid units. Another attempt to promote rigidity, and consequently, free volume was the exploitation of the roof-shaped triptycene unit, which revealed encouraging results [34,35]. Moreover, it has been established that a further effective way to improve gas separation performance is to insert bulky side groups, which create high free volume. The use of rigid, bulky groups provides better size discrimination ability, thus, greater selectivity [36]. Numerous researchers studied the effect of bulky groups, e.g., tert-butyl or trimethylsilyl, introduced in different polymers such as polyimide [36,37], polynorbornene [38] and polyacetylene [39,40,41]. The studies showed that the incorporation of bulky substituents increases free volume and, thus, the gas permeability coefficient.

Despite the abundant studies of PIM on gas separation, there remains a paucity of research on water transport properties since it is intricate in using the hydrophobic nature of PIM-1 to pass water molecules through the micropores [17]. However, water scarcity is a crucial problem directly affecting our living standards; therefore, a strong need has emerged to develop energy-efficient and ecofriendly methods of water separation [42]. Conventional water separation methods are forward osmosis, reverse osmosis, ultrafiltration, nanofiltration and membrane distillation (MD). Considering the low operating temperature, MD is a promising and energy-efficient technique in which only water vapor molecules are able to pass through the hydrophobic porous membrane [43]. In comparison to the transport of ideal gases, water vapor transport could be discussed from a different point of view due to its unique transport mechanism stemming from water molecule-polymer and water-water molecules interactions. Up to now, far too little investigation has been done on water vapor transport in PIMs.

This study contributes to the aforementioned works by the investigation of the influence of aliphatic side-chain group size and shape of new anthracene maleimide polymers on their properties. It was expected that the roof shape of anthracene-maleimide could provide a rigid and non-coplanar structure that restricts chain packing of the polymer [35]. In this study, new anthracene-maleimide monomers were employed for the synthesis of homopolymers and copolymers with spiro-bisindane comonomers, which were further studied for gas transport properties. The change in aliphatic chain length (methyl-, ethyl- and *n*-propyl-), change in group geometry (propyl-, *i*-propyl- and *t*-butyl-) and difference between cycloaliphatic and aromatic cycles (cyclohexyl- and phenyl-) are the main subjects able to reveal a trend between the change in the structure of the side group and the transport parameters of gaseous penetrants. To this end, the thermal treatment of homopolymer methyl-100 thick film was conducted to establish retro-Diels-Alder rearrangement of the homopolymer. In a previous research article, Khan et al. [44] studied the effect caused by different aromatic substituents on the gas properties of polymers.

To our knowledge, there is a lack of published data correlating regular polymer structure change in substituted anthracene maleimides with gas and vapor transport properties. It is further anticipated that the introduction of the maleimide group into the monomer would increase the polarity of the polymer and subsequently improve water vapor permeability coefficient.

## 2. Materials and Methods

### 2.1. Materials

5,5′,6,6′-tetrahydroxy-3,3,3′,3′-tetramethyl-1,1′-spirobisindane (TTSBI, 98%) was purchased from ABCR GmbH (Karlsruhe, Germany). 2,3,5,6-tetrafluoro-terephthalonitrile (TFTPN, 99%) was obtained from Lanxess (Cologne, Germany). TFTPN was sublimated twice at 70 °C under vacuum before use. Cyclohexyl maleimide (98%) was purchased from TCI (Portland, OR, USA). *t*-Butyl maleimide was purchased from Specific Polymers (Castries, France). Boron tribromide (BBr_3_, 99%) was obtained from Acros Organics (Geel, Belgium). Potassium carbonate (K_2_CO_3_, 99%), methyl maleimide (98%), dimethyl acetamide (DMAc, 99%) and dichloromethane (DCM, 99%) were purchased from Alfa Aesar (Kandel, Germany). All other commercially available reagents were obtained from Merck (Darmstadt, Germany) and were used without further treatment.

### 2.2. Monomer Synthesis

#### 2.2.1. 2,3,6,7-Tetramethoxy-9,10-Dibutylanthracene

At first, 1,2-dimethoxybenzene (43.4 g, 314 mmol) was added to 100 mL concentrated H_2_SO_4_ (70% in water) at 0–5 °C. Afterward, pentanal (46 g, 534 mmol) was added dropwise over a period of 1 h. The viscous violet mixture was stirred at room temperature for 4 days. The highly viscous blackish reaction mixture was poured into a mixture of 2 L ethanol-water (1:1), and the precipitate was collected by filtration. Subsequently, washing with acetone and drying at 70 °C in vacuum yielded 13 g of light yellow powder (20%), Mp. 325 °C. ^1^H-NMR (500 MHz, CDCl_3_): δ (ppm): 0.98 (*t*, 6 H), 1.50 (m, 4 H), 1.72 (m, 4 H), 3.35 (m, 4 H), 3.98 (s, 12 H), 7.33 (s, 4 H).

#### 2.2.2. 2,3,6,7-Tetrahydroxy-9,10-Dibutylanthracene

Boron tribromide (BBr_3_) (15.8 g, 63 mmol) dissolved in 90 mL dichloromethane (DCM) was added slowly to a cooled mixture of 10 g 2,3,6,7-tetramethoxy-9,10-dibutylanthracene (24 mmol) in 265 mL DCM under argon atmosphere. After the complete addition of BBr_3_, the reaction was stirred for approximately 18 h and was terminated by the addition of 60 mL of water. After pouring an excess of water, the mixture was stirred for 2 days. The precipitate was filtered, washed with water and dried at 60 °C in vacuum to give 11 g grey powder (98%), Mp. 220 °C. ^1^H-NMR (500 MHz, DMSO-d6): δ (ppm): 1.01 (*t*, 6 H), 1.54 (m, 4 H), 1.61 (m, 4 H), 3.18 (*t*, 4 H), 7.32 (s, 4 H), 9.38 (s, 4 H).

#### 2.2.3. Methyl-Comonomer

A mixture of 1 g 2,3,6,7-tetrahydroxy-9,10-dibutylanthracene (2.8 mmol) and 0.35 g methyl maleimide (3.1 mmol) in 19 mL o-xylene was refluxed at 150 °C for 5 h. The brown reaction suspension was poured into cyclohexane. Filtration and drying at 65 °C under vacuum gave 1.20 g of a dark brown powder, (92%), ^1^H-NMR (500 MHz, DMSO-d6): δ (ppm): 1.13 (*t*, 6 H), 1.70 (m, 6 H), 1.94 (m, 2 H), 2.11 (m, 2 H), 2.42 (s, 3 H), 2.67 (m, 2 H), 3.18 (s, 2 H), 6.58 (s, 2 H), 6.69 (s, 2 H), 8.60 (s, 2 H), 8.70 (s, 2 H).

#### 2.2.4. Ethyl-Comonomer

Ethyl-comonomer was synthesized by the same method as the methyl-comonomer, using ethyl maleimide instead of methyl maleimide to give a dark brown solid, (83%),^1^H-NMR (500 MHz, DMSO-d6): δ (ppm): 0.41 (*t*, 3 H), 1.10 (*t*, 6 H), 1.70 (m, 6 H), 1.91 (m, 2 H), 2.06 (m, 2 H), 2.64 (m, 2 H), 2.97 (m, 2 H), 3.12 (s, 2 H), 6.57 (s, 2 H), 6.68 (s, 2 H), 8.56 (s, 2 H), 8.68 (s, 2 H).

#### 2.2.5. Propyl-Comonomer

Propyl-comonomer was synthesized by the same method as the methyl-comonomer, using propyl maleimide instead of methyl maleimide to give a dark brown solid, (74 %), ^1^H-NMR (500 MHz, DMSO-d6): δ (ppm): 0.41 (*t*, 3H), 0.88 (m, 6H), 1.10 (*t*, 6H), 1.70 (m, 2H), 1.93 (*t*, 2H), 2.11 (m, 2H), 2.67 (m, 4H), 2.96 (*t*, 2H), 3.16 (m, 2H), 6.59 (s, 2H), 6.69 (s, 2H), 8.57 (s, 2H), 8.69 (s, 2H).

#### 2.2.6. *i*-Propyl-Comonomer

A solution of 3 g isopropyl amine (50.7 mmol) in 54 mL THF was added dropwise to a solution of 5.5 g maleic anhydride (55.8 mmol) in THF, and then the mixture was stirred for 5 h at room temperature. The reaction mixture was filtrated and residual THF was removed under reduced pressure to obtain 66.4 g 4-oxo-4-(propan-2-ylamino)but-2-enoic acid as a white powder, (80%), ^1^H-NMR (500 MHz, DMSO-d6): δ (ppm): 1.18 (m, 6H), 4.05 (m, H), 6.18 (d, H), 6.25 (d, H), 8.39 (s, H), 15.49 (s, H).

Sixty-six grams of 4-oxo-4-(propan-2-ylamino)but-2-enoic acid (38.17 mmol) was dissolved in 46 mL acetic acid, and the solution was immersed in an oil bath at 80 °C. The reaction mixture was kept stirring for 24 h at 80 °C and was terminated by pouring into water. The resulting emulsion was then extracted with *n*-pentane. A colorless liquid product of isopropyl maleimide was obtained after pentane removal under reduced pressure, (21%), ^1^H-NMR (500 MHz, DMSO-d6): δ (ppm): 1.37 (d, 6 H), 4.25 (m, H), 7.03 (s, 2 H).

Further, the *i*-propyl-comonomer was synthesized by the same method as the methyl-comonomer, using isopropyl maleimide instead of methyl maleimide to give a dark brown solid, (81%), ^1^H-NMR (500 MHz, DMSO-d6): δ (ppm): 0.83 (d, 6 H), 1.10 (*t*, 6 H), 1.68 (m, 6 H), 1.91 (m, 2 H), 2.08 (m, 2 H), 2.65 (m, 2 H), 3.08 (s, 2 H), 3.76 (m, H), 6.59 (s, 2 H), 6.68 (s, 2 H), 8.57 (s, 2 H), 8.69 (s, 2 H).

#### 2.2.7. *t*-Butyl-Comonomer

*t*-Butyl-comonomer was synthesized by the same method as the methyl-comonomer, using *t*-butyl maleimide instead of methyl maleimide to give a dark brown solid, (95%), ^1^H-NMR (500 MHz, DMSO-d6): δ (ppm): 1.07 (m, 15 H), 1.63 (m, 6 H), 1.90 (m, 2 H), 2.04 (m, 2 H), 2.56 (m, 2 H), 2.95 (s, 2 H), 6.59 (s, 2 H), 6.66 (s, 2 H), 8.57 (s, 2 H), 8.66 (s, 2 H).

#### 2.2.8. Cyclohexyl-Comonomer

Cyclohexyl-comonomer was synthesized by the same method as the methyl-comonomer, using cyclohexyl maleimide instead of methyl maleimide to give a dark brown solid, (91%), ^1^H-NMR (500 MHz, DMSO-d6): δ (ppm): 0.85 (d, 2 H), 1.11 (m, 10 H), 1.68 (m, 10 H), 1.92 (m, 2 H), 2.09 (m, 2 H), 2.67 (m, 2 H), 3.10 (s, 2 H), 3.40 (m, 1 H), 6.59 (s, 2 H), 6.69 (s, 2 H), 8.58 (s, 2 H), 8.69 (s, 2 H).

#### 2.2.9. Phenyl-Comonomer

This comonomer and the associated homo- and copolymers were obtained by a procedure published elsewhere [44].

### 2.3. Polymer Synthesis

#### 2.3.1. PIM-1 Synthesis

PIM-1 was synthesized by using the fast synthesis method reported elsewhere [45,46,47]. A slight modification of the reported method was employed in this study. A three-necked round bottom flask was charged with TTSBI (20 mmol) and TFTPN (20 mmol) and dissolved in 65 mL dimethylacetamide (DMAc) under argon atmosphere. Addition of K_2_CO_3_ (44 mmol) turned the reaction mixture from orange to yellow. The reaction flask was immediately immersed into a preheated oil bath (150 °C). The gradual addition of diethylbenzene (DEB) (15 mL) prevented the precipitation of PIM-1 and promoted the formation of a polymer with higher molecular weight. The reaction mixture was stirred for 30 min and was quenched by precipitation in methanol. After filtration, the solid was dried at 80 °C in vacuum. The dried polymer was dissolved in CHCl_3_ and re-precipitated in methanol. The solid was collected by filtration and dried under high vacuum at 80 °C to give the final product as a yellow solid (90%). The apparent molecular weight (*M_w_*) of the purified polymer and dispersity (*Ð*) were determined by size-exclusion chromatography, giving an apparent average molecular weight of *M_w_* = 132.5 kg/mol and *Ð* = 4.9.

#### 2.3.2. Synthesis of Homo- and Copolymers

Copolymers were synthesized by polycondensation of new comonomers/TTSBI (1 mmol/1 mmol) and TFTPN (2 mmol) using the earlier described procedure similar to that of PIM-1 synthesis. Copolymers will be referred to as methyl-/ethyl-/propyl-/*i*-propyl-/*t*-butyl-/cyclohexyl-/phenyl-50 where the suffix −50 indicates the comonomer/TTSBI mol ratio in the copolymers (e.g., methyl-50 consists 50 mol% TTSBI and 50 mol% comonomer unit).

Homopolymers were synthesized by polycondensation of new comonomers and TFTPN (equimolar amount) using a similar procedure to that of PIM-1. Homopolymers will be referred to as methyl-/ethyl-/propyl-/*i*-propyl-/*t*-butyl-/cyclohexyl-/phenyl-100 where the suffix −100 stands for the comonomer/TTSBI mol ratio in the homopolymers (e.g., methyl-100 consists of 100 mol% comonomer unit). A slight modification of the abovementioned PIM-1 synthesis was carried out for homopolymerization, which is that, instead of the addition of DEB, DMAc was used in 2-fold excess volume, and the reaction time was increased for an additional 1 h.

### 2.4. Film Preparation

Thick polymer films were obtained by casting from a solution containing 3–5% wt. of polymer dissolved in CHCl_3_. The homogenous solution was obtained by stirring for at least 2 h followed by casting into a leveled Teflon^®^ circular dish. The solution in the dish was covered with a glass lid, and the system was purged gently with an argon stream for 24 h to evaporate CHCl_3_ at room temperature. After solvent evaporation, the thick film was removed from the Teflon^®^ surface and further dried for 24 h in vacuum at 80 °C. The thickness of the obtained films was in the 60–80 µm range as determined by a digital micrometer Deltascope FMP10 (Helmut Fisher GmbH, Sindelfingen, Germany).

### 2.5. Methods

#### 2.5.1. Size-Exclusion Chromatography (SEC)

For SEC measurements, a column combination (precolumn-SDV-linear, SDV-linear and SDV 102 nm with inner diameter = 4.6 mm and length = 53 cm, Polymer Standard Service GmbH, Mainz, Germany) was used at a flow rate of 1.0 mL min^−1^ running CHCl_3_ as eluent at 30 °C. A combination of refractive index (RI) and ultraviolet (UV) detectors were used for concentration detection. Polystyrene standards of different molecular weights were used for the evaluation of apparent weight average molecular weight (*M_w_*) and dispersity index (*Ð*) of the prepared polymers.

#### 2.5.2. ^1^H Nuclear Magnetic Resonance (^1^H-NMR)

NMR spectra were collected on a Bruker AV500 NMR spectrometer (Bruker Biospin, Ettlingen, Germany) operating at a field of 7T (500 MHz) using a 5 mm ^1^H/^13^C TXI probe and a sample temperature of 298 K. ^1^H^1^ NMR spectra were recorded applying a 10 ms 90° pulse.

#### 2.5.3. Thermal Gravimetric Analysis (TGA)

TGA measurements were performed using TGA/DSC 2 Mettler-Toledo, Giessen, Germany). The analysis was performed under argon flow (20 mL/min) in the temperature range 30 to 800 °C at a heating rate of 5 K min^−1^.

#### 2.5.4. Thermogravimetric Analysis Coupled with Fourier Transform Infrared Spectroscopy (TG-FTIR)

The evolved gases were measured using a thermogravimetric analyzer coupled with an FT-IR spectrometer Nicolet iS50 (Thermo Scientific, Darmstadt, Germany), which was recorded in the spectral range of 400–4000 cm^−1^ with a resolution of 4 cm^−1^ and an average of 8 scans. All measurements were performed in the temperature range 30 to 800 °C at a heating rate of 5 K min^−1^.

#### 2.5.5. Fourier Transform Infrared Spectroscopy (FTIR)

FTIR spectra were collected on an ALPHA FTIR spectrometer (Bruker Optics, Bremen, Germany) in attenuated total reflectance mode (ATR, diamond crystal). The measurements were done at ambient temperature in a spectral range of 400 to 4000 cm^−1^ with a resolution of 4 cm^−1^ and an average of 64 scans.

#### 2.5.6. Density Measurement

The density measurements were performed based on the buoyancy method, as described elsewhere [45]. FC-770, Fluorinert (3M, Saint Paul, MN, USA) was used as an immersion liquid because it has the least affinity to the polymers under study and would not diffuse into the free volume of the polymer during measurement. The density was determined according to Equation (2):

(2) $$\rho =\frac{\chi (\rho_{o}-\rho_{l})}{\chi -\beta }+\rho_{l}$$

where, χ is weight of the sample in air, *β* is weight of the sample in displaced liquid, $\rho_{o}$ is density of fluorinated liquid (1.79 g cm^−3^ determined at conditions of the experiment) $\rho_{l}$ is density of air (0.001 g cm^−3^).

#### 2.5.7. Determination of Fractional Free Volume (FFV)

The fractional free volume (FFV) of polymers under study was determined by the combination of experimental and molecular modeling data, which were applied to Equation (3):

(3) $$FFV=\frac{V-1.3V_{w}}{V}$$

where *V* is the polymer specific volume, and *V_w_* is the specific van der Waals volume obtained by molecular modeling [48]. The specific volume of the polymers was obtained from density measurements. Van der Waals volume can be obtained using the group contribution method by Bondi [49], summarized by van Krevelen [50]. In the current work, a molecular modeling approach was utilized where a polymer chain consisting of 3 monomeric units was equilibrated by geometry optimization functions of HyperChem 8.0 (Hypercube Inc, Gainesville, FL, USA) software. After full molecule optimization, the QSAR function of HyperChem was called out and molecular weight and van der Waals volume of a single monomeric unit was derived. Modeling of the chain consisting of 3 monomeric units allowed for the reduction of the end group effect on the final result. The validity of volume calculation was confirmed by a comparison of results with that obtained in Materials Studio (BIOVIA, San Diego, CA, USA) [46]. Since HyperChem is easier to use for this simple task, it was decided to model all polymers using this software.

#### 2.5.8. Gas Transport Properties Measurement

The gas transport properties of synthesized polymers were investigated by the “time-lag” method utilizing the constant volume/variable pressure approach on the facility described elsewhere [51]. H_2_, N_2_, O_2_, CH_4_ and CO_2_ were considered as permanent gases at conditions of experiment: 30 °C, 1000 mbar feed pressure, 0–10 mbar permeate pressure and 238 cm^3^ constant permeate volume. The effective membrane area was 1.15 cm^2^. Transport properties of H_2_O vapor were investigated as well; the feed pressure, in this case, was ca. 60% of saturated vapor pressure but, despite the fact that water vapor is very close to the saturation point, the data were treated the same way as for the other gases, not taking into account the fugacity coefficient. Each gas was applied to the thoroughly evacuated polymer film 3 times, and average values were taken. In case the difference in values was bigger than 5%, the measurement was repeated. The time lag measurements were conducted without any further treatment right after film preparation to minimize the physical aging effect.

The permeability (P), diffusion (D) and solubility (S) coefficients and ideal permeability selectivity ($\alpha_{x/y}$) for gases *x* and *y* were determined from the linear part of the “time-lag” curve by the following equations:

(4) $$P=DS=\frac{V_{p}l\left(P_{p2}-P_{p1}\right)}{ART\Delta t\left(p_{f}-\left(p_{p2}+p_{p1}\right)/2\right)}$$

(5) $$D=\frac{l^{2}}{6\theta }$$

where $V_{p}$ is the constant permeate volume (cm^3^), l is the film thickness (cm), A is the effective area of the membrane (cm^2^), R is the universal gas constant (8.314 J mol^−1^ K^−1^), *T* is the absolute temperature of experiment (°C), Δt is the time for the permeate pressure change from $p_{p1}$ to $p_{p2}$ (s), $p_{f}$ is the feed pressure (cmHg) and θ is the time lag(s). The accuracy of the gas transport parameters depends strongly on the film preparation quality for the diffusion coefficient and on the pressure applied to the o-ring sealing of the measurement cell for the permeability coefficient. The thickness of studied films was in the range of 80 µm with a thickness spread ±2 µm which gives ca. 2.5% error for the diffusion coefficient determination. The experimental error of permeability coefficient determination can be considered as 4% for the case of film area of 1.15 cm^2^ exposed to penetrant and 1 mm diameter Viton^®^ O-ring used for sample sealing. Other parameters, e.g., accuracy of pressure and temperature sensors, can be considered insignificant in comparison to these two parameters.

## 3. Results and Discussions

### 3.1. Comonomer Synthesis

**Figure 1:**
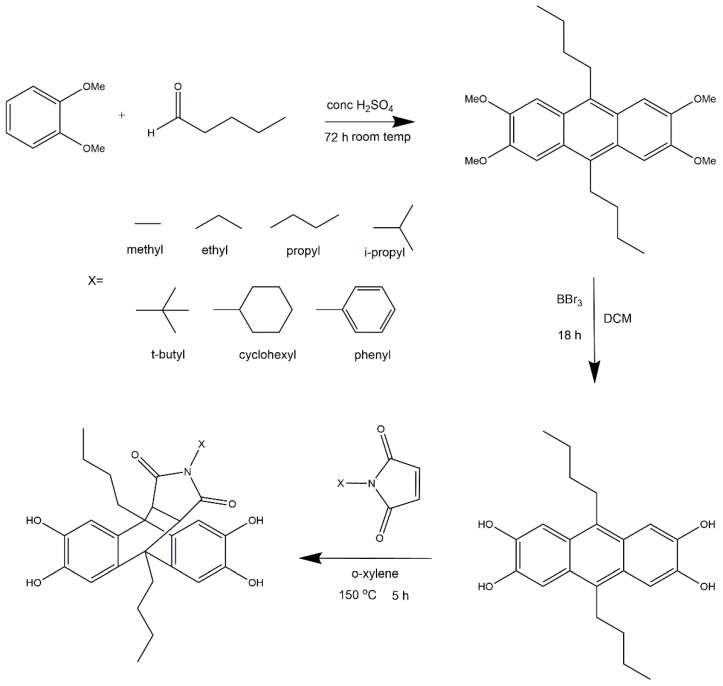
Synthesis route of the new comonomers.

Comonomers were synthesized following the multi-stage synthesis route as shown in Figure 1, inspired by the procedure published by Khan et al. [44]. However, a slight modification of the reported procedure was employed in this study. The first step in this process is the condensation reaction of 1,2-dimethoxybenzene and pentanal to obtain tetramethoxy anthracene. The decision to use pentanal was based on the improved solubility of the reaction product. The hydroxylation of the tetramethoxy anthracene intermediate was the next step to obtain the tetrahydroxy anthracene derivative. After the successful conversion of tetramethoxy to tetrahydroxy anthracene, a Diels-Alder reaction was carried out using maleimides, which contain different substituents attached to the nitrogen atom. In comparison to the procedure established by Khan et al. [44], the anthracene-maleimide comonomers were obtained in a shorter reaction time but with a higher yield in this study.

**Figure 2:**
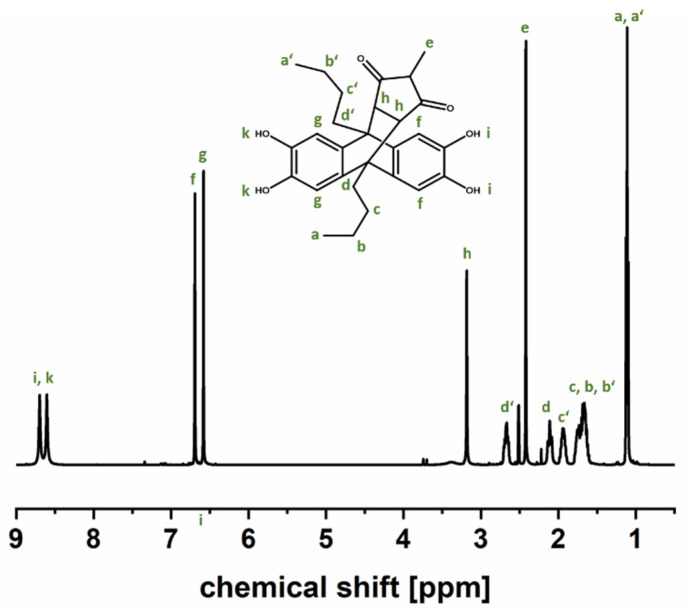
^1^H NMR spectrum of the methyl-comonomer.

The success of all comonomer syntheses was shown by ^1^H NMR measurements (Figure S1). As an example, the ^1^H NMR spectrum of the methyl-comonomer is shown in more detail in Figure 2. The spectrum exhibits two singlets at 8.69 and 8.61 ppm, assigned to the hydroxyl protons, and two singlets at 6.67 and 6.59 ppm, corresponding to the aromatic protons. The signal split is due to the asymmetrical structure of the monomer, which arises from the formation of a “roof-like structure” and the tilting of this group towards one side of the anthracene backbone. The two characteristic protons, which appear after successful Diels-Alder reaction and the formation of the “roof-like structure”, are present at 3.18 ppm, whereas one sharp singlet appears at 2.41 ppm assigned to the aliphatic protons of the *N*-methyl substituent. The protons attributed to the butyl groups appear in the range of 2.74 to 1.09 ppm. The other comonomers show similar spectra regarding the monomer backbone protons (aromatic, hydroxyl, butyl and maleimide groups linked to anthracene backbone). The residual spectra show that variations and signals overlap depending on the size of the substituent (Figure S1).

**Figure 3:**
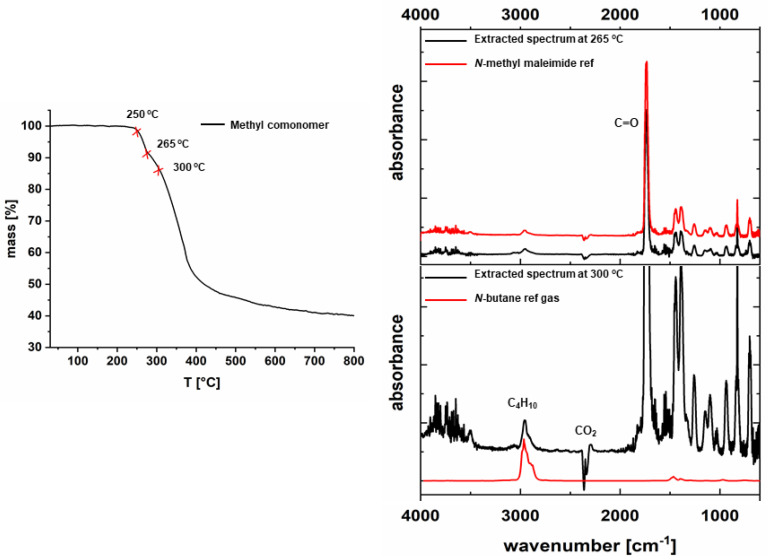
TG-FTIR spectra of methyl-comonomer.

TGA was carried out to determine the decomposition temperature of the comonomers since polymerization takes place at 150 °C, and a retro-Diels-Alder reaction was reported for aromatic substituents [44]. TG-FTIR analysis was performed in the temperature range from 30 °C to 800 °C. Figure 3 depicts that the methyl-comonomer remains stable up to approximately 250 °C and degradation of the monomer takes place in two clearly visible steps. In order to identify the early degradation products, gas-phase FTIR spectra were obtained at 265 °C and 300 °C. The gas-phase spectra show FTIR bands at 2953 cm^−1^ related to C-H stretching vibration of the methyl group, 1737 cm^−1^ due to C=O stretching and 697 cm^−1^ related to the H-C=C-H bending vibration of methyl maleimide, which is in very good accordance to a gas-phase spectrum, collected from a TG-FTIR measurement of neat *N*-methyl maleimide and verifies the occurrence of a retro-Diels-Alder reaction as described for aromatic substituents [44]. Additionally, the extracted spectrum obtained for the sample taken at 300 °C shows the appearance of *n*-butane. According to these results, we can infer that the decomposition of the maleimide group takes place prior to the comonomer backbone decomposition. In addition to the methyl-comonomer, also ethyl and *t*-butyl-comonomers show the same behavior regarding the lower retro Diels-Alder temperature. On the other hand, the decomposition of the comonomer backbones of propyl, *i*-propyl, cyclohexyl and phenyl comonomers overlaps with that of the retro Diels-Alder reaction.

### 3.2. Polymer Synthesis

**Figure 4:**
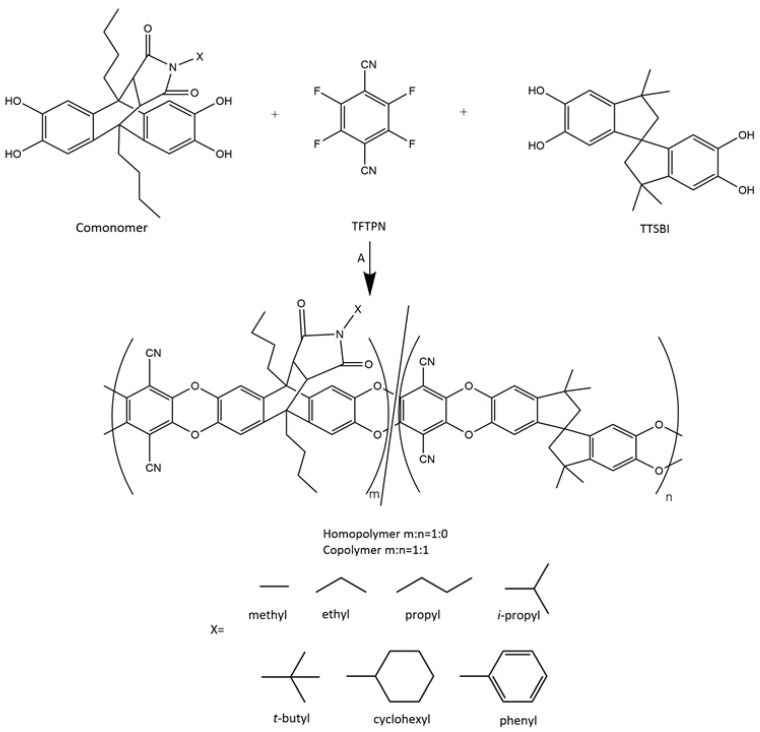
Synthesis of homopolymer and copolymer, A: reagents and conditions DMAc, DEB (for copolymer), K_2_CO_3_, 150 °C, 30–60 min.

After successful monomer synthesis, all comonomers were polymerized in the next step. The homo- and copolymers were synthesized by the polycondensation of comonomers (Figure 4) with TFTPN and comonomer/TTSBI (molar ratio 1/1) with TFTPN, respectively, by using a procedure similar to PIM-1 synthesis with slight modification [44,47,52]. DEB addition takes place only in the synthesis of copolymers, whereas for homopolymer synthesis, it is not necessary. This discrepancy might be related to the different solubility of homopolymer and copolymer solutions [25]. PIM-1 usually starts to precipitate when the number of repeating units reaches a certain degree of polymerization. Since copolymers also contain the repeating units of PIM-1, a similar situation might occur in this study.

**Figure 5:**
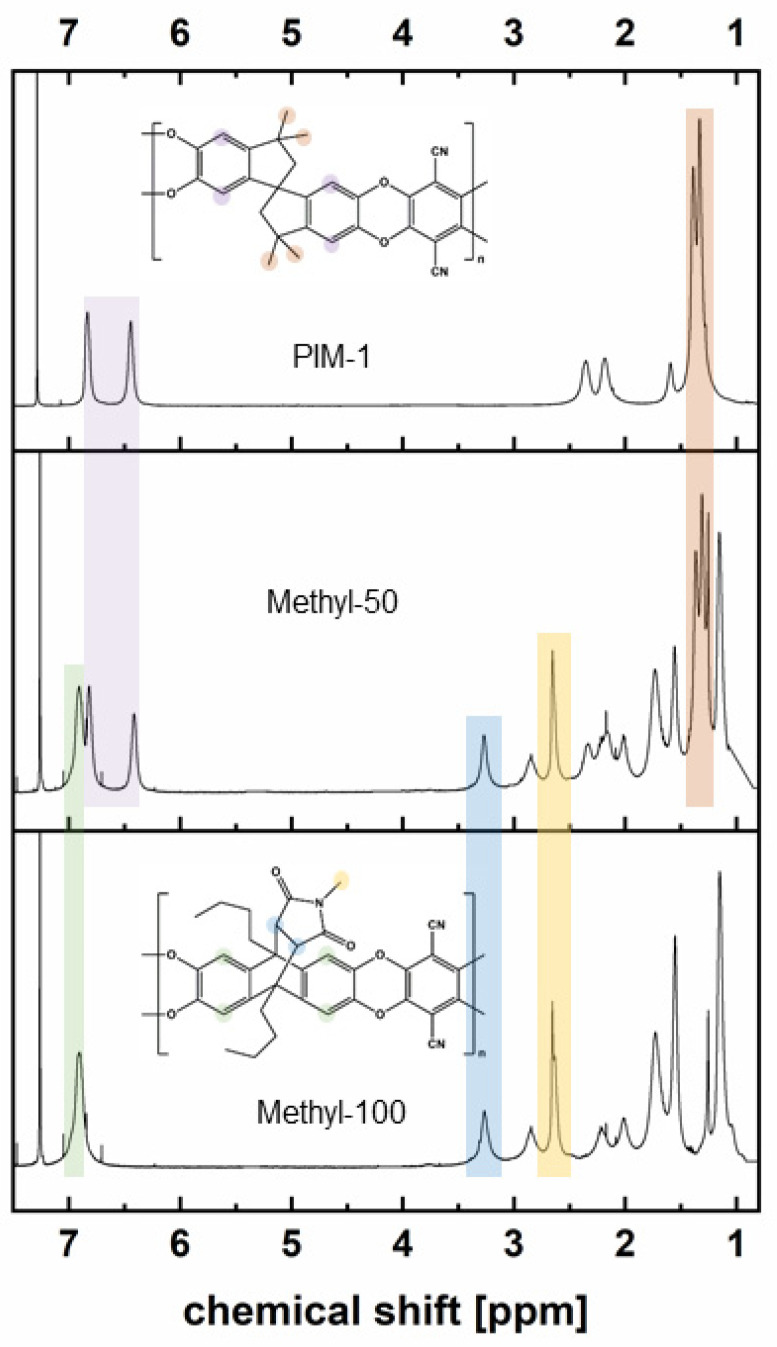
^1^H-NMR spectra of PIM-1, methyl-50 and methyl-100.

All homo- and copolymers were characterized with respect to their apparent molar masses and dispersity by gel permeation chromatography (GPC) and their compositions by ^1^H-NMR spectroscopy (Table S1). Figure 5 depicts ^1^H-NMR spectra of methyl-50 and methyl-100 in comparison to PIM-1. The peak seen at 6.45 ppm for PIM-1 and methyl-50 spectra arises from the aromatic protons of TTSBI. The characteristic signal of the comonomer corresponding to the roof-shaped maleimide proton was seen at 3.25 ppm, which indicates the stability of comonomers throughout the polymerization conditions. The integration of allocated peaks of methyl-50 proved that the composition of the copolymer (methyl comonomer/TTSBI) is close to equimolar, as expected. Table S1 shows the entire polymer composition calculations based on the ^1^H-NMR spectra.

**Figure 6:**
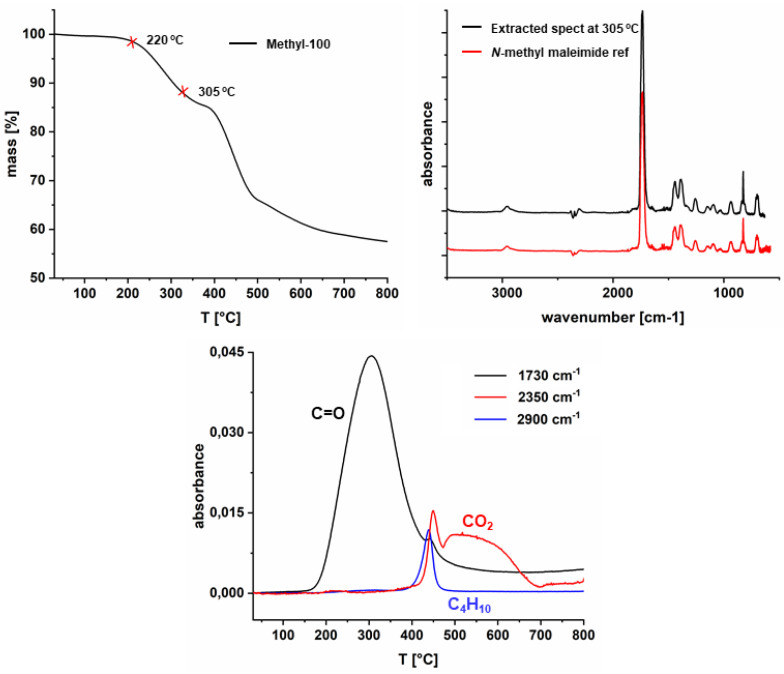
TG-FTIR spectrum of methyl-100.

TG-FTIR analysis was carried out to determine the retro Diels-Alder temperature of homopolymers. TGA curves for all homopolymers exhibit two distinct onset decomposition temperatures (Figure S3). As can be seen from Figure 6, the onset temperature of the first decomposition step of methyl-100 is at approximately 220 °C. It arises from breaking the maleimide moiety, and the second decomposition around 400 °C corresponds to the decaying of the polymer backbone. The evolved gas analysis was conducted at 305 °C, and the extracted spectrum was compared with the reference *N*-methyl maleimide gas-phase spectrum. Additionally, peak height profiles of the carbonyl band at 1730 cm^−1^ and evolved gases (CO_2_ and *n*-butane) revealed that similar to the monomers, retro-Diels-Alder reaction (in the case of the polymers) takes part before butyl group degradation. Moreover, no CO_2_ was detected below 350 °C, leading to these inferences: (1) the peak evolved around 300 °C which means, that only retro-Diels-Alder takes part and there is no further degradation from maleimide or group disintegration; (2) the peaks evolving above 400 °C correspond to CO_2_ (2350 cm^−1^) and C_4_H_10_ (2900 cm^−1^) caused by the degradation of the polymer backbone. These features indicate that the retro Diels-Alder reaction occurs at lower temperatures than the decomposition of the polymer backbone chain and does not overlap with the butane release correlated with decomposition of the polymer backbone.

**Figure 7:**
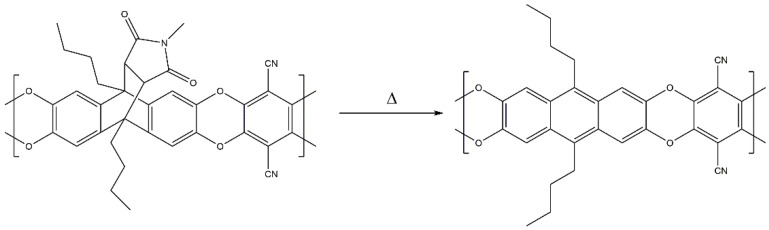
Expected thermal conversion of methyl-100 at 250 °C.

**Figure 8:**
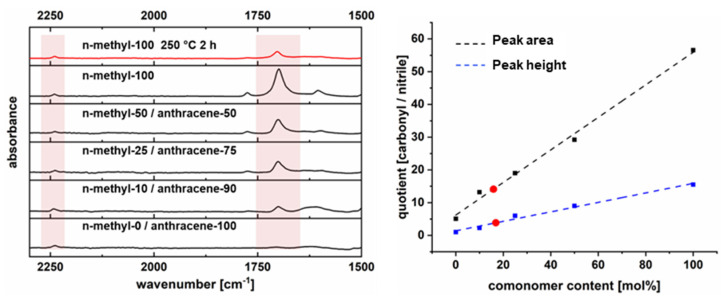
Quantification of conversion by means of FT-IR.

These findings offer further opportunity for a post-thermal treatment of polymer films, which would lead to materials with structures shown in Figure 7. Although it is not a focus of this study, the effect of the thermal conversion was tested on the example of methyl-100. In order to quantify the extent of the thermal conversion, four additional polymers were synthesized. These polymers consist of 2,3,6,7-tetrahydroxy-9,10-dibutylanthracene (synthesis described earlier) and methyl-comonomer in different molar ratios (0:1, 0.9:0.1, 0.75:0.25, 0.5:0.5, 1:0) and were used for establishing a calibration graph by means of FT-IR (Figure 8). After synthesis, it was observed that all materials are not soluble in common solvents (THF, CHCl_3_) that are normally used for the processing of PIMs. This means that the thermal conversion of the materials does not only change the structure, but also the solubility properties of the polymers.

After thermal treatment at 250 °C for 2 h in a nitrogen atmosphere, the polymer film was measured by means of FT-IR (ATR). The ratios of the integrated peak areas and the peak height of the nitrile and carbonyl groups were used for quantification. Both methods gave approximately 16% residual methyl maleimide. Therefore, 84% of the maleimide groups were removed at 250 °C, while the polymer film remained mechanically stable. The optical images of methyl-100 films before and after thermal treatment can be seen in Figure S4.

In this study, differential scanning calorimetry measurement (DSC) of homo- or copolymers was not conducted since the decomposition temperature is lower than PIM-1. It is known that the glass transition temperature (T_g_) of PIM-1 cannot be detected before its thermal decomposition using conventional DSC [53].

### 3.3. Gas and Water Vapor Transport Properties

All polymer films prepared from the synthesized polymers were transparent and mechanically stable enough to be sealed in the measurement cell of the “time-lag” facility with a Viton^®^ O-ring. Films were placed into the experimental facility after the same, shortest possible time after solvent evaporation in order to keep all polymers at a similar state in respect to possible physical aging. No additional experiments on thick polymer film aging were carried out since this topic was investigated earlier [54] and was not intended for the current study. Before this, the gas transport measurement films were evacuated to the state when the permeate pressure sensor did not show any signs of pressure increase due to the desorption of volatile impurities from the polymer film. After the evacuation step, films were exposed to penetrants in the following sequence: H_2_, N_2_, O_2_, CH_4_, CO_2_ and H_2_O. The gas was taken from the gas line or from the liquid water container into the feed pressure vessel and brought to the temperature of the thermostated part of the “time-lag” facility. The gas permeability, diffusion and solubility coefficients of gases and vapor for films of PIM-1, copolymers and homopolymers are presented in Tables S2–S4. In the further discussion, gas transport parameters will be analyzed mostly on the examples of CH_4_ and CO_2_ as penetrants with a significant difference in kinetic diameters and state in respect to critical pressure and temperature, and water vapor as an example of a penetrant very close to the condensation under experimental conditions.

#### 3.3.1. Gas Transport Properties of Homopolymers and Copolymers

An initial objective of using different substituted maleimides was to investigate the effect of aliphatic groups of different sizes and shapes on gas and water vapor transport properties. The change in linear aliphatic chain length (methyl-, ethyl- and propyl-), change in group geometry (propyl-, *i*-propyl- and *t*-butyl-) and difference between cycloalkyl and aromatic groups (cyclohexyl- and phenyl-) were the main subjects able to reveal a trend between change in the structure of the side group and transport parameters of gaseous penetrants in synthesized polymers.

**Figure 9:**
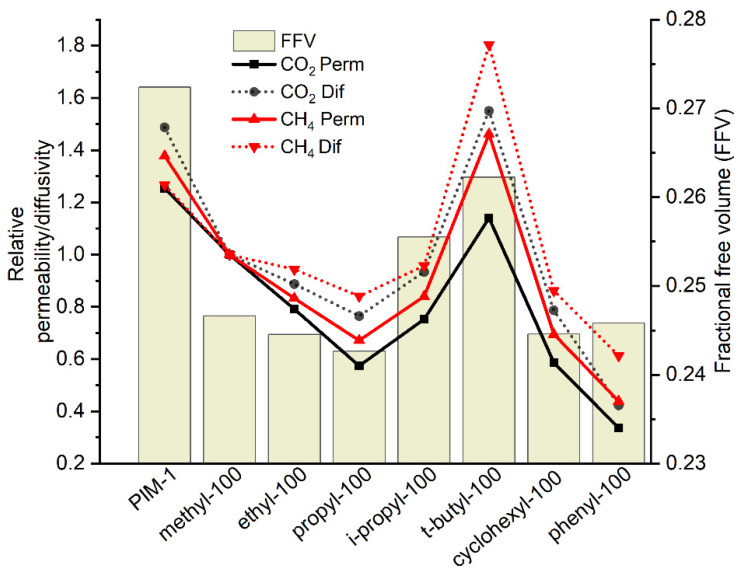
CO_2_ and CH_4_ relative permeability relative diffusivity and FFV of homopolymers. (Relative permeability or diffusivity: homopolymer gas permeability or diffusivity are normalized to methyl-100 gas permeability or diffusivity.)

In comparison to PIM-1, the newly synthesized homopolymers exhibit lower single gas permeability coefficients. Figure 9 shows that there is a gradual reduction of CH_4_ and CO_2_ permeability coefficients in the line of the methyl-, ethyl- and propyl- substituted homopolymers. When the linear alkyl chain splits, as in the cases of *i*-propyl-100 and *t*-butyl-100 polymers, forming so-called “bulky” side groups [39], permeability starts to increase in comparison to linear side groups. This result is also supported by further observations. By introducing a third methyl group into the alkyl chain, as seen in *t*-butyl-100, there is an improvement in CO_2_ and CH_4_ permeability, which, in the case of CH_4_ in *t*-butyl-100, became even higher than that of PIM-1. This can be associated with an increase of the fractional free volume (FFV) for *i*-propyl-100 and *t*-butyl-100 polymers in comparison to linear side group polymers. It is interesting to observe that the FFV of *t*-butyl-100 polymer is still lower than in the case of PIM-1, but the CH_4_ diffusion coefficient is nearly 40% higher while the diffusion coefficient of CO_2_ is very similar to PIM-1. This fact can only be related to differences in the structure of the free volume in these two polymers, giving a lower molecular sieving effect for *t*-butyl-100. Introduction of spacious, “bulky” side groups, as in the case of *t*-butyl-100, where flexibility of the side chain is restricted, leads to a desirable change of the FFV even when the size of the group is relatively small in comparison to the size of the monomeric unit. This phenomenon is in accordance with previous studies indicating that the introduction of bulky side groups leads to inefficient chain packing, thus resulting in higher FFV [36,55]. For a better FFV analysis, CH_4_ is a good choice considering its biggest kinetic diameter among all other studied penetrants (H_2_O: 2.65 Å; H_2_: 2.89 Å; CO_2_: 3.3 Å; O_2_: 3.46 Å; N_2_: 3.64 Å; CH_4_: 3.8 Å). Figure 9 exhibits that CH_4_ and CO_2_ diffusion coefficients of homopolymers follow the same pattern. It can be seen that there is a gradual reduction of the relative diffusion coefficient along with side chain length increase. A possible explanation for this might be that the linear side chains, due to their flexibility, probably fill the free volume formed by packing of macromolecular chains but do not create additional free volume, as in the case of *i*-propyl and *t*-butyl groups, causatively reducing diffusion coefficients for both CH_4_ and CO_2_. As previously mentioned, in cases of *i*-propyl-100 and *t*-butyl-100, as soon as the rigid, bulky side group is introduced into the polymer structure, the diffusion coefficient increases as a consequence of the rise in FFV. Introduction of the cyclic substituent into the polymer structure does not bring benefits nor for FFV value or for gas transport properties of CH_4_ or CO_2_. Permeability coefficients of gases in cyclohexyl-100 are very similar to that in propyl-100, and significantly lower than for the homopolymer with bulky substitutes. Due to the possible boat and chair molecular conformations, the cyclohexyl substituent can be considered as a flexible side group, which can effectively fill the free volume elements. This implication is suggested by the FFV value of the homopolymer, which for cyclohexyl-100, is almost the same as for propyl-100 and much lower than those of *i*-propyl-100 or *t*-butyl-100. Surprisingly, CH_4_ and CO_2_ permeability of phenyl-100 is lower compared to other homopolymers while possessing almost the same FFV of homopolymers with linear side chains. A possible explanation of this finding might be that the phenyl side group can rotate freely and possibly interact with aromatic rings of the main chain by π-π stacking, consequently not increasing the FFV of the polymer as in the case of other spacious groups. In this manner, the phenyl substituent might be addressed as a viably flexible side chain rather than called a rigid, bulky group [44].

Gas transport properties of copolymers are discussed by considering the effect of the introduction of 50 mol% of comonomer units into the PIM-1 polymer backbone. It must be reckoned that the main disadvantage of the polycondensation mechanism for copolymer synthesis is its limitation to control the distribution of the monomeric units along the polymer chain and the formation of oligomers in contrast to addition type polymerizations [47,56]. Consequently, copolymerization of PIM-1 and anthracene maleimide comonomer units leads, probably, to a random distribution of the different units along the polymer chain if the reactivity of their functional groups is similar.

**Figure 10:**
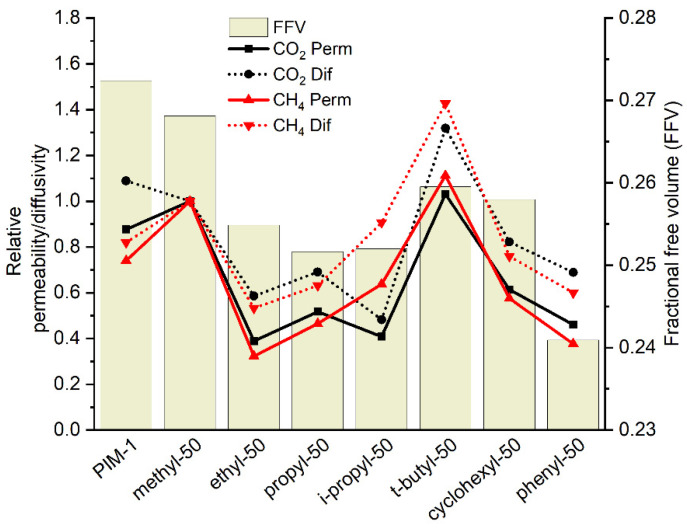
CO_2_ and CH_4_ relative permeability relative diffusivity and FFV of copolymers. (Relative permeability or diffusivity: copolymer gas permeability or diffusivity are normalized to methyl-50 gas permeability or diffusivity.)

In comparison to PIM-1, methyl-50 and *t*-butyl-50, copolymer presence increased gas permeabilities (Table S2). Figure 10 depicts that the CO_2_ permeability in methyl-50 is increased by 14%, while in *t*-butyl-50, it is increased by 18% in comparison to PIM-1. Regarding CH_4_ permeability, methyl-50 exhibits 35% and *t*-butyl-50 exhibits 50% higher values compared to PIM-1. It can therefore be assumed that the introduction of methyl or *t*-butyl anthracene maleimide structures into PIM-1 at a 1:1 molar ratio helps to increase permeability by ca. 20% compared to PIM-1, accompanied by an ca. 20% decrease in CO_2_/CH_4_ selectivity.

However, the line for permeability of copolymers does not show the same behavior when compared to that for homopolymers. Figure 10 demonstrates that the FFV of copolymers does not follow the same trend, which was attributed to the linear and bulky side group effect, as discussed in the homopolymer case. An unanticipated finding is that methyl-50 has a higher FFV than *t*-butyl-50. One can conclude that the aforementioned random copolymerization results in the mixing of geometrically different PIM-1 and substituted maleimide monomers play an important role in polymer chain packing, making it especially inefficient in the case of methyl-50 polymer and resulting in higher free volume and permeability coefficients. Unfortunately, it is difficult to have a clear knowledge of the comonomer distribution within the copolymers. Herein, further research is required to evaluate reaction kinetics for maleimide and spirobisindane units and to understand whether copolymerization is truly random or block formation is possible. The ambiguity of the reaction kinetics could lead to a non-uniform arrangement of the polymer packing, hereby causing the formation of an irregular structure of the FFV.

Furthermore, N_2_, H_2_ and O_2_ transport data of homopolymers and copolymers can be found in the supporting information. Figures S5 and S6 clearly show that N_2_, H_2_ and O_2_ permeability and diffusivity lines follow the same trend as those of CO_2_ and CH_4_ for homopolymer and copolymer.

#### 3.3.2. Water Vapor Transport Properties of Homo- and Copolymers

The outcome of water transport in synthesized homo- and copolymers indicates that the introduction of an anthracene maleimide derivative with different side chains plays a significant role in water transport through the polymer matrix. Numerous publications report that imide-based membranes are extensively used for water separation since they possess good mechanical stability, high thermal resistance and high water vapor permeability [57,58,59]. It is known that the imide group increases the polarity of the molecule and provides an environment for water molecules to form hydrogen bonding [60,61,62]. Hence, the introduction of this group into the polymer chain would lead to an improvement in the interaction between the water molecule and the polymer. This estimation is supported by the results of water transport in homopolymers displayed in Table 1.

**Figure 11:**
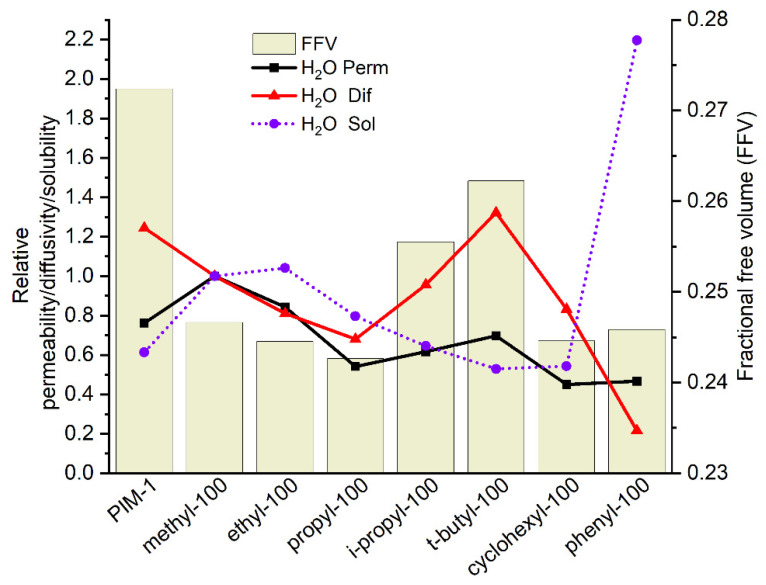
H_2_O relative permeability relative diffusivity, relative solubility and FFV of homopolymers. (Relative permeability or diffusivity or solubility: homopolymer water permeability or diffusivity or solubility are normalized to methyl-100 water permeability or diffusivity or solubility.)

In comparison to PIM-1, methyl-100 and ethyl-100 show higher water permeabilities in accordance with the expectation. Among the substituents, the methyl-imide derivative is assumed to give the most polar comonomer since it has the shortest alkyl side chain, which influences the molecule’s polarity, and therefore restricts the interaction of water with the macromolecule less than larger aliphatic substituents. Previous studies show that larger alkyl groups inversely act on water permeability independent of membrane application, e.g., gas separation or pervaporation [63,64]. Along with the increase in alkyl side chain length, water vapor permeability decreases, as can be observed in the line of methyl-100, ethyl-100, and propyl-100 polymers. The reason for this can be attributed to the fact that a longer aliphatic chain reduces the affinity of the imide side-chain group toward H_2_O, most probably due to increased hydrophobicity and thus effective blocking of the imide ring vicinity for water molecules. This consideration is confirmed by the absence of the aforementioned bulky group effect on CH_4_ and CO_2_ permeability for the case of water permeability. Table 1 exhibits that water permeability of *i*-propyl-100 and *t*-butyl-100 increases insignificantly contrary to cases for CO_2_ and CH_4_ as a result of replacing the linear side chain by bulkier groups accompanied with a significant increase in free volume. Cyclic side groups also did not bring any significant improvement to water permeability. It is obvious to evaluate the permeability data in conjunction with the diffusion and solubility coefficients and fractional free volume of polymers in accordance with the solution-diffusion model. Taking into account both the solubility and diffusivity effects in Figure 11, the water vapor permeability of homopolymers with alkyl side chains mostly follows changes of solubility, while the diffusion coefficient gradually decreases in the line from methyl to propyl side groups along with reduction of free volume. When the linear side group is changing to more spacious *i*-propyl- and *t*-butyl- substituents, the water diffusion coefficient increases following the change of the FFV. At the same time, the water solubility coefficient decreases and reaches minimum values for *t*-butyl and cyclohexyl substituents. In comparison to the methyl case, both of these factors, i.e., changes in solubility and diffusion coefficients, result in relatively low water vapor permeability coefficients, which can be explained only by blocking the access of water molecules to the imide moiety by the bulky substituent.

**Figure 12:**
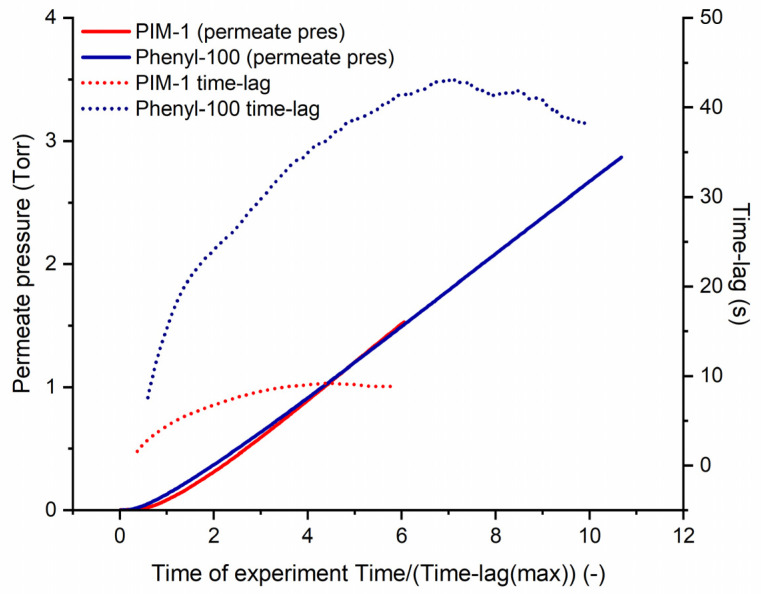
Time-lag curve analysis of PIM-1 and phenyl-100.

To understand water transport through polymers better, it is worth mentioning some specific phenomena regarding the penetrant behavior. Generally, when there is an affinity of water penetrant to the polymer (high hydrophilicity), the interaction of water-polymer leads to polymer swelling, which is also called plasticization [65], or even dissolution. On the other hand, if the polymer is rather hydrophobic, the water-water interaction can be more prominent than the water-polymer interaction. As a result of prevailed penetrant-penetrant association, water molecules can form clusters due to hydrogen bonding [65,66,67]. These two phenomena significantly influence water diffusivity and, consequently, permeability through the membrane in different ways. Kelkar and Paul showed that plasticization causes the polymer chains to move freely on a segmental length scale, thus increasing water diffusivity [68]. However, when the molecule clustering effect overrides the plasticization, water molecule clusters increase in average size; subsequently, the apparent diffusivity of water decreases. Several studies reported the importance of the clustering effect on water transport [67,68,69,70,71,72]. In this current study, the transport of water molecules in polymers is in compliance with the solution-diffusion model without strong evidence of specific interactions between the water molecule and polymer. Therefore, the abovementioned clustering of water molecules or polymer swelling are probably not preponderant phenomena—a result based on our “time-lag” experimental results, with the exception of the phenyl-100 case. More concrete, the result of the gas transport parameters revealed a strong deviation of the water vapor solubility coefficient of phenyl-100. Further analysis of the experimental data has clearly shown the presence of at least two time-lags on the curve of permeate pressure increase during the “time-lag” experiment. The presence of at least two time-lags is obviously observed by the analysis curves derived from the experimental data when the time-lag value is plotted with the development of time. In detail, a certain number of experimental points were used to plot a tangential line by using the “least squares method”. In the present case, 37 points were taken since this was the minimum number of points suitable for the analysis of the water permeation curve through phenyl-100, while the same number of points was used for other polymers: PIM-1, methyl-100, *t*-butyl-100 and cyclohexyl-100. The data set of 37 points was moved along the water permeation curve (y_1_-axis), and the corresponding values of the tangential line intersection with time (y_2_-axis) were plotted along with the time-lag development with the time of the experiment (x-axis). As can be seen from Figure 12, the experimental curves for PIM-1 and phenyl-100 in the range of 1–3 time lags have a slight difference in shape. However, the curves of the time-lag development have a significant difference. The red dotted line in Figure 12 corresponding to PIM-1 shows a proper time-lag curve, which reaches its maximum value of 9.5 s at 4.4 time-lags (42.2 s after experiment started). On the other hand, the curve for phenyl-100 exhibits the presence of at least two time-lags which occur at ca. two time-lags (89.6 s of experiment) and at 7.16 time-lags (320 s of experiment), where the curve reaches its maximum of 44.8 s. Remarkably, the analysis was carried out according to the maximum time-lag values observed from which diffusion and the corresponding solubility coefficients are derived. These diffusion and solubility coefficients can be considered as “effective” coefficients, indicating the behavior of the polymer during the water vapor permeation and thus the collection of the water vapor in the permeate side of the facility. Further analysis of obtained results should be carried out according to, e.g., Jansen et al. [73].

As mentioned before, the increase of the permeate pressure curves was analyzed for several polymers. Table 2 shows the comparison of the moments when the time-lag values of polymers reach their maximum. It can be seen from the table data that the maximum time-lag of the polymers was reached at 4.5 ± 0.4 time-lags after the experiment started. However, phenyl-100 is an exceptional case in which the maximum time-lag is reached at 7.16. A possible explanation for this might be the presence of at least two diffusion coefficients for water vapor in phenyl-100 attributed to the formation of water molecule clusters in free volume elements of this polymer. These findings raise intriguing questions regarding the difference between phenyl-100 and the other homopolymers such as methyl-100 or cyclohexyl-100, which lead to this discrepancy. Similar to findings in a previous study [73,74] referring to the diffusion of alcohol vapors through Teflon^®^ AF and Hyflon^®^ AD, the presence of several diffusion coefficients can be explained by the water molecules clustering in the free volume of hydrophobic polymers. Moreover, if the phenyl substitution leads to a specific interaction between the functional groups of the polymer located on the “surface” of the free volume elements, it could explain the confounding information stored in the “time-lag” curve. In relation to that, prior studies have noted the importance of the interaction of water with aromatic rings [75,76,77]. Levitt and Perutz claimed that aromatic groups might act as hydrogen bond acceptors due to their electrostatic interaction arising from carbon and hydrogen atoms of aromatic rings [75]. Moreover, Vojislavlijevic et al. [76] emphasized the position of the water molecule accommodated near an aromatic ring, highlighting the OH-π electron interactions between water and aromatic rings. Answers to these questions should be examined by investigation of phenyl-100 in the presence of water vapor by solid-state NMR or other spectroscopic methods able to reveal either change in water self-diffusion or shift in peaks characteristic for the bonds able to interact with water. It is also suggested to develop a detailed analysis tool of the time-lag data similar to the one reported by Friess et al. [74]. Further investigation of water vapor transport in synthesized polymers or their analogs is also interesting since phenyl-50 copolymer does not show any evidence of multiple diffusion coefficients, the solubility coefficient of water is in the usual range as in, e.g., PIM-1.

**Figure 13:**
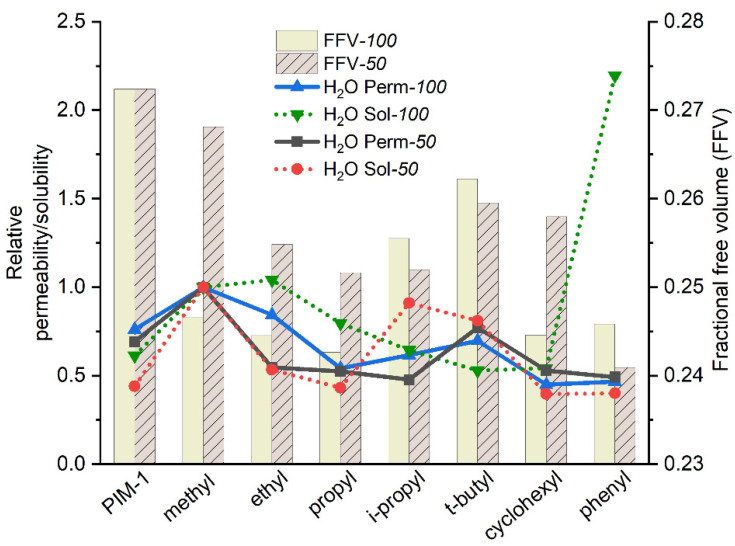
H_2_O relative permeability and relative solubility and FFV of copolymer and homopolymers. (Relative permeability/ solubility: polymer water permeability or solubility are normalized to methyl- water permeability or solubility.)

The water vapor permeability trend for copolymers is consistent with the trend of water solubility. Figure 13 depicts that the shift in water permeability follows nearly the same pattern with solubility, except for *i*-propyl-50. It can be seen that methyl-50 has higher water vapor permeability than PIM-1, similar to the case of methyl-100. The aforementioned effect of affinity toward water might be the reason for this behavior. Since imide with methyl side group is assumed to be the most polar among all substituents, the observation of the highest water vapor permeability and solubility fits the assumption.

Surprisingly, the water solubility peak seen in phenyl-100 does not demonstrate a similar leap in phenyl-50. Random distribution of monomer units could be responsible for such a discrepancy, as was discussed before. Taking into consideration that such random chain packing exists, the spirobisindane monomer unit, which is more hydrophobic, might hinder or stultify the effect of the phenyl side group on water interaction. Whether the increment of water vapor permeability is attributed to FFV or substituents affinity to water vapor remains a matter of question. Furthermore, the FFV of the copolymer is mostly higher than those of the homopolymer, as expected. This is attributed to the contorted structure resulting from the spirobisindane unit of PIM-1, which causes enhancement on interconnected voids. However, the FFV of *i*-propyl-50 and *t*-butyl-50 are slightly higher than those of related homopolymers. Such behavior might occur due to the high impact of bulky side groups. Overall, it is important to point out the fact that a random distribution of copolymer units plays a prominent role in the arrangement of FFV and also polymer-molecule affinity.

Gas transport properties of methyl-100 after thermal treatment (methyl-100-TT) were tested by a time-lag measurement (Table S5). The results revealed a significant permeability and diffusivity drop of all single gases and water vapor. These results are likely due to the physical aging of the polymer films because of their exposure to high temperature. Nevertheless, it would be misleading to compare the permeability and diffusion coefficients of polymer films before and after thermal treatment, since they were not exposed to the same conditions. Taking this fact into account, one interesting finding can still be sorted out of these results—the water vapor solubility of methyl-100-TT increased more than two folds compared to methyl-100. This observation may support the hypothesis that was mentioned before regarding the aromatic ring-water molecule affinity. After thermal treatment, the polymer structure was rearranged, and the roof-shaped maleimide moiety was removed by the formation of the third aromatic ring in the backbone. It can be therefore assumed that water solubility escalated due to the increase of aromatic groups. However, since the vapor diffusion coefficient decreased, possibly due to the collapse of FFV arising from physical aging, water vapor permeability does not show an increase, as does the solubility. To develop a full picture of the gas transport properties of thermally treated polymer films, additional studies are needed.

## 4. Conclusions

The aim of the present research was to examine the effect of different alkyl chains on gas and water vapor transport. Anthracene maleimide-based comonomers with different alkyl side chains were successfully synthesized by the Diels-Alder reaction. ^1^H-NMR and TG-FTIR characterizations of comonomers were properly established. TG-FTIR results of methyl, ethyl and *t*-butyl comonomers revealed that the decomposition temperature of the maleimide unit occurs prior to that of the anthracene unit. Consequently, the outcome provided the further possibility to employ the retro-Diels Alder rearrangement of homopolymers. New comonomers were further used for homopolymerization with TFTPN and then used for copolymerization in the combination of TFTPN and TTSBI monomer units. All polymers were characterized via ^1^H-NMR, GPC and TG-FTIR. ^1^H-NMR spectra of copolymers confirmed the 1:1 molar ratio of the comonomer TTSBI. TG-FTIR analysis of methyl-100 revealed that the retro-Diels Alder reaction of the maleimide group occurs earlier than the decomposition of the homopolymer backbone. Based on this finding, post-thermal treatment of methyl-100 and gas transport measurements were tested to set an example of structure rearrangement by retro-Diels Alder reaction.

Gas and water vapor transport properties of all newly synthesized polymers were established by the time-lag method. Methyl-50 and *t*-butyl-50 exhibit higher CO_2_ and CH_4_ permeability than PIM-1. The distinguished effect of linear alkyl and bulky side chains on the fractional free volume was discussed, and it was shown that bulky side groups lead to a better permeability of these gases. The influence of the anthracene maleimide unit with different side groups revealed that the affinity of the side group strongly affects water vapor transport. Methyl-100 and ethyl-100 show higher water vapor permeability and solubility than PIM-1 and other homopolymers. However, phenyl-100 showed a jump in water vapor solubility with a low diffusion coefficient, which is attributed to the formation of the water molecule clusters located in the free volume elements of the polymer. This research provides a framework for the exploration of employment of the new polymer series with anthracene maleimide for water vapor transport studies in a broader concept of water treatment applications. Nevertheless, it is recommended that further research be undertaken in order to validate the water vapor transport parameters of these glassy polymers.

## Figures and Tables

**Table 1:** Water vapor permeability of homopolymers and copolymers.

	PIM-1	Methyl-100	Ethyl-100	Propyl-100	*i*-Propyl-100	*t*-Butyl-100	Cyclohexyl-100	Phenyl-100
Water permeability (Barrer *)	79,300	110,000	87,800	56,300	64,100	72,500	46,800	48,600
		**Methyl-50**	**Ethyl-50**	**Propyl-50**	***i*-Propyl-50**	***t*-Butyl-50**	**Cyclohexyl-50**	**Phenyl-50**
Water permeability (Barrer *)		114,500	62,700	60,100	54,800	88,500	60,600	56,400

* 1 Barrer = 1 × 10^−10^ (cm^3^(STP) cm cm^−2^ s^−1^ cmHg^−1^ = 3.348 × 10^−16^ mol m m^−2^ s^−1^ Pa^−1^.

**Table 2:** Comparison of the moments where the time-lags of polymers reach their maximum values.

Polymer	Time [Time-Lags]
PIM-1	4.44
Methyl-100	4.49
*t*-Butyl-100	4.90
Cyclohexyl-100	4.17
Phenyl-100	7.16
Phenyl-50	3.80

## Data Availability

The data presented in this study are available on request from the corresponding author.

## Supplementary Materials

The following are available online at https://www.mdpi.com/article/10.3390/polym14010119/s1, Figure S1: ^1^H NMR spectra of all comonomers; Figure S2: TGA curves of comonomers; Figure S3: TGA curves of homopolymers; Figure S4: Optical images of methyl-100 before and after thermal treatments; Figure S5: N_2_, O_2_, H_2_ transport properties of homopolymers; Figure S6: N_2_, O_2_, H_2_ transport properties of copolymers; Table S1: Molecular weight data of polymers by GPC and polymer compositions calculated by ^1^H NMR; Table S2: Permeability coefficients of PIM-1, homo- and copolymers determined at 30 °C; Table S3: Diffusion coefficients of PIM-1, homo- and copolymers determined at 30 °C; Table S4: Solubility coefficient of PIM-1, homo- and copolymers determined at 30 °C; Table S5: Gas transport properties of methyl-100-TT* determined at 30 °C.
