# Supplementary material for: Investigation of the Side Chain Effect on Gas and Water Vapor Transport Properties of Anthracene-Maleimide Based Polymers of Intrinsic Microporosity
Source: Polymers (Basel). 2021 Dec 29;14(1):119. doi: 10.3390/polym14010119 (PMC8747615; doi:10.3390/polym14010119)
Supplement: Supplementary file 1 [file polymers-14-00119-s001.zip › polymers-1503713-supplementary.pdf]

polymers

MDPI

Supplementary Information

Investigation of the side chain effect on gas and water vapor transport properties of anthracene-maleimide based polymers of intrinsic microporosity

Esra Caliskan[1], Sergey Shishatskiy[1], Silvio Neumann[1], Volker Abetz[1,2], Volkan Filiz[1,] *

[1] Helmholtz-Zentrum Hereon, Institute of Membrane Research, Max-Planck-Str. 1, 21502 Geesthacht, Germany
[2] University of Hamburg, Institute of Physical Chemistry, Martin-Luther-King-Platz 6, 20146 Hamburg, Germany

* Correspondence: volkan.filiz@hereon.de; Tel.: +49-41-5287-2425

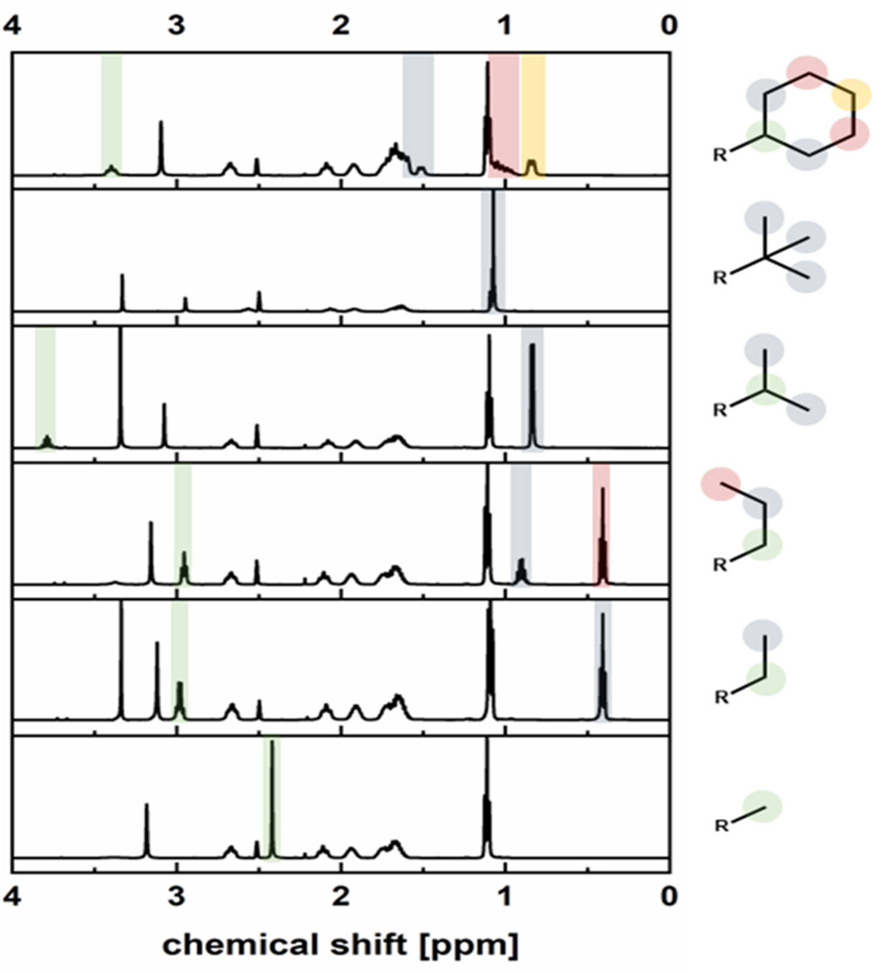

Figure S1. ^{1}H NMR spectra of all comonomers

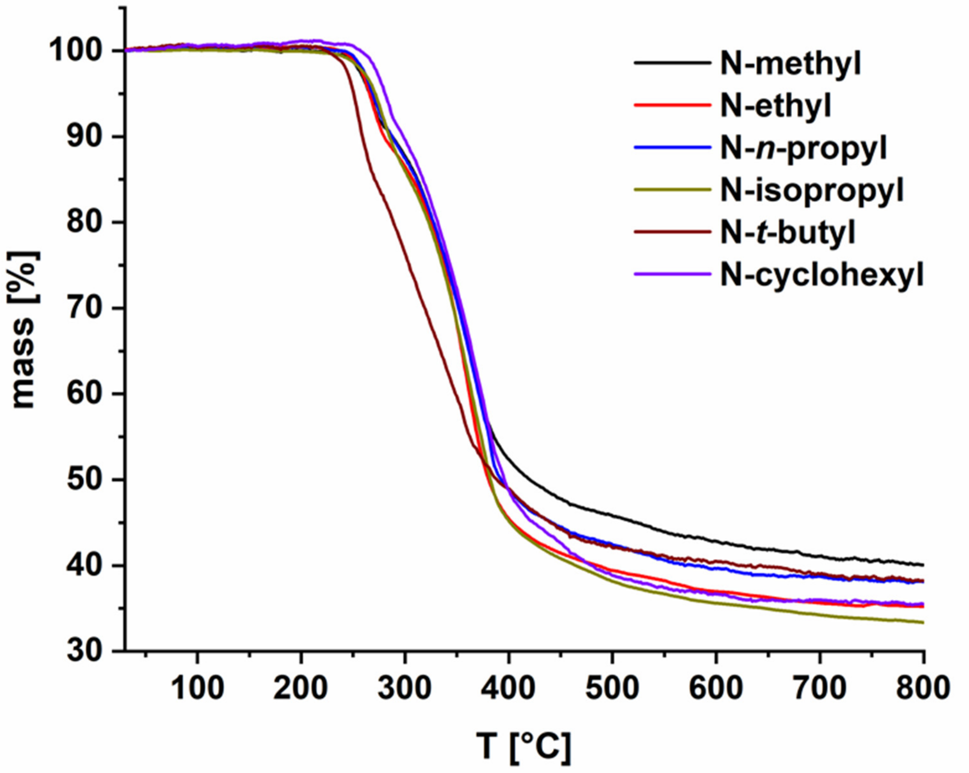

Figure S2. TGA curves of comonomers

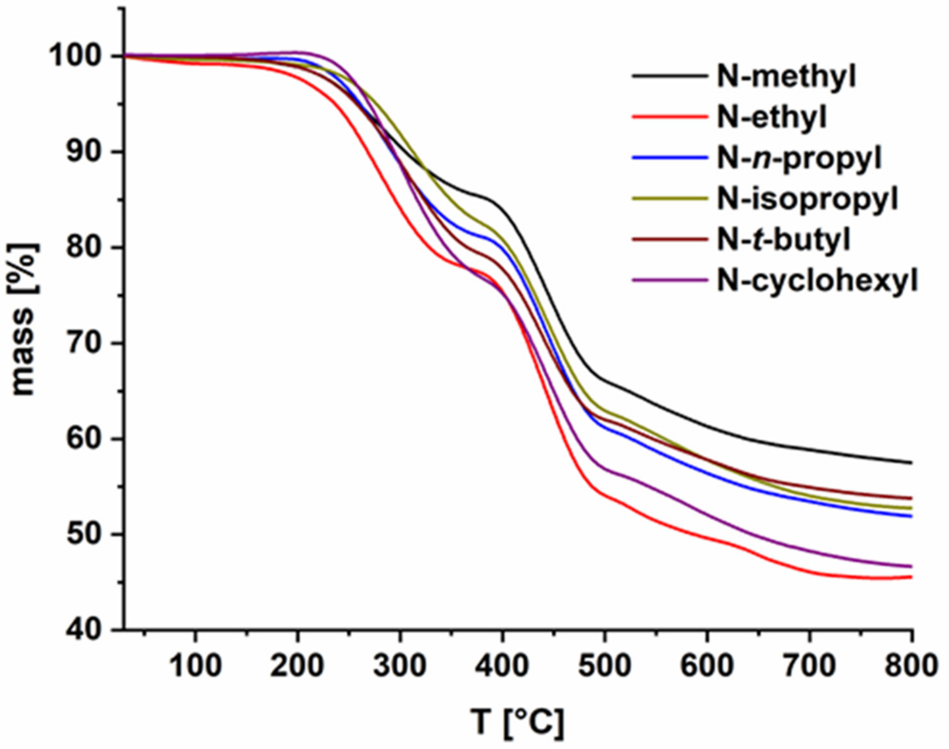

Figure S3. TGA curves of homopolymers

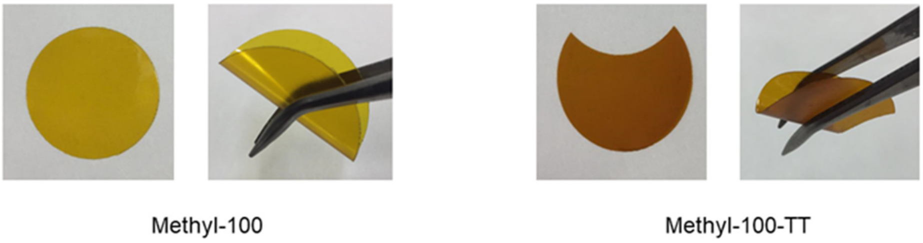

Figure S4. Optical images of methyl-100 before and after thermal treatments

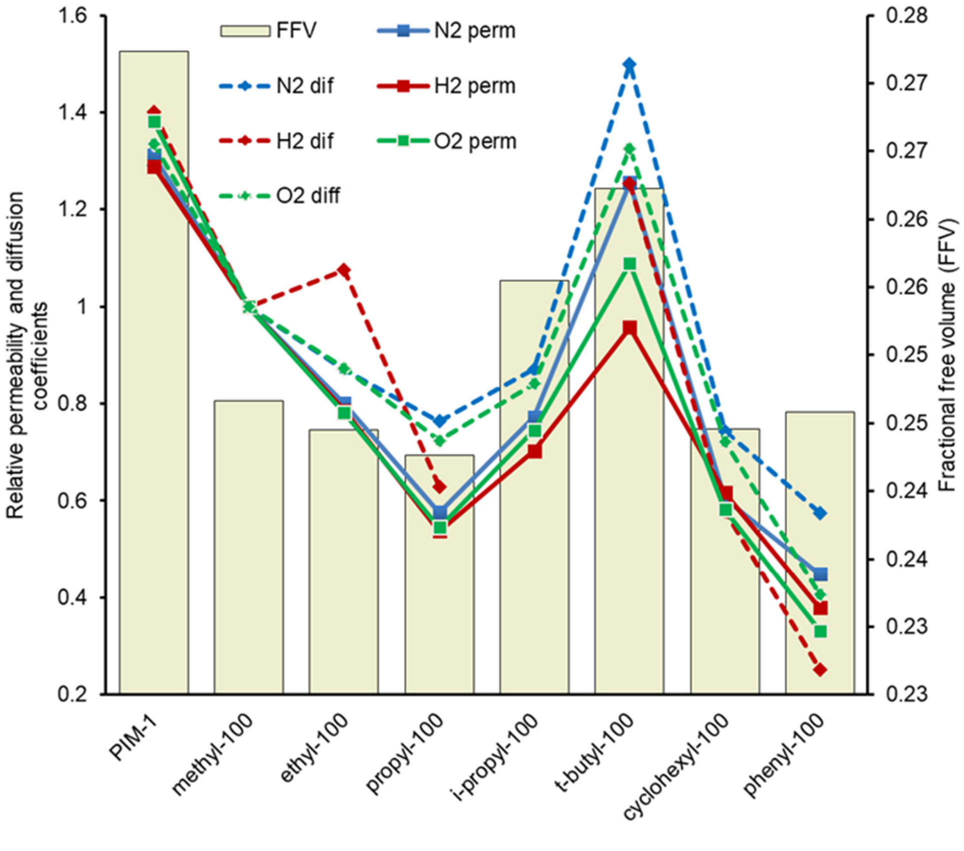

Figure S5. N_2, O_2, H_2 transport properties of homopolymers

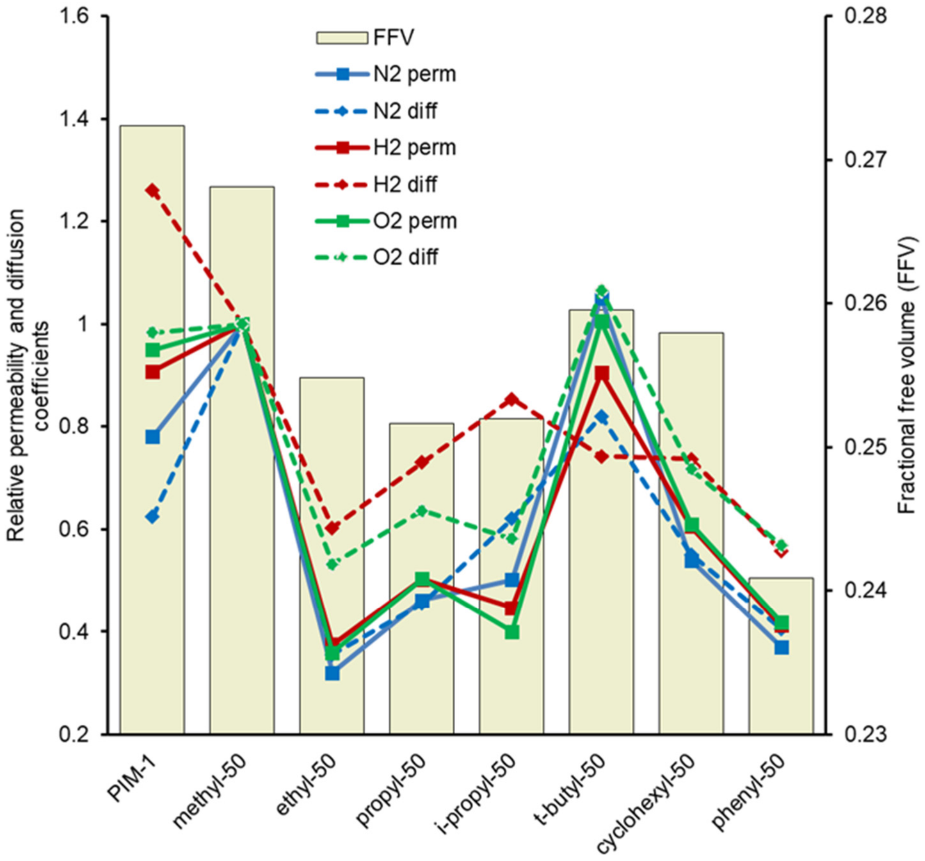

Figure S6. N_2, O_2, H_2 transport properties of copolymers

Table S1. Molecular weight data of polymers by GPC and polymer compositions calculated by ^{1}H NMR

	M_w (kg/mol) *	Đ	Comonomer amount (%) **
methyl-100	45.9	4.35	-
methyl-50	85.5	4.24	49
ethyl-100	52.9	3.53	-
ethyl-50	68.5	3.49	50
propyl-100	38.2	3.72	-
propyl-50	75.1	3.75	49
i-propyl-100	61.3	3.73	-
i-propyl-50	98.0	4.32	48
t-butyl-100	78.4	3.67	-
t-butyl-50	97.1	5.27	48
cyclohexyl-100	45.7	3.51	-
cyclohexyl-50	63.5	3.44	48
phenyl-100	78.3	3.43	-
phenyl-50	76.3	3.86	47

Table S2. Permeability coefficients of PIM-1, homo- and copolymers determined at 30°C

	**Permeability (Barrer)**					
	**H_2**	**N_2**	**O_2**	**CO_2**	**CH_4**	**H_2O**
PIM-1	2450	300	950	6120	475	79300
Homopolymer						
methyl-100	2160	300	735	4990	445	110000
ethyl-100	1500	185	540	3860	290	87800
propyl-100	1020	135	375	2800	230	56300
i-propyl -100	1340	180	510	3680	290	64100
t-butyl -100	1820	290	750	5570	505	72500
cyclohexyl -100	1170	140	400	2860	240	46800
phenyl-100	720	105	230	1640	150	48600
Copolymer						
methyl-50	2700	390	995	6980	640	114500
ethyl -50	1010	125	360	2720	210	62700
propyl -50	1360	180	500	3610	300	60100
i-propyl -50	1200	190	400	2500	300	54800
t-butyl -50	2440	410	1010	7200	710	88500
cyclohexyl-50	1630	210	610	4280	370	60600
phenyl-50	1110	145	420	3210	240	56400

Table S3. Diffusion coefficients of PIM-1, homo- and copolymers determined at 30°C

	**Diffusion coefficient (*10⁷)**					
	**H_2***	**N_2**	**O_2**	**CO_2**	**CH_4**	**H_2O**
PIM-1	640	7.3	21.1	8.1	2.5	8.5
Homopolymer						
methyl-100	460	5.7	15.8	5.4	2.0	6.8
ethyl-100	690	4.9	13.8	4.8	1.9	5.5
propyl-100	290	4.3	11.4	4.2	1.7	4.6
i-propyl-100	-	4.9	13.3	5.1	1.9	6.5
t-butyl-100	570	8.5	21.0	8.4	3.6	9.0
cyclohexyl-100	270	4.2	11.4	4.3	1.7	5.7
phenyl-100	120	3.3	6.4	2.3	1.2	1.5
Copolymer						
methyl-50	510	11.7	21.5	7.4	3.1	5.5
ethyl-50	310	4.2	11.4	4.4	1.6	5.4
propyl-50	370	5.3	13.7	5.1	1.9	6.6
i-propyl-50	430	7.3	12.5	3.9	2.3	2.2
t-butyl-50	380	9.6	22.9	9.8	4.4	5.2
cyclohexyl-50	370	6.5	15.4	6.1	2.3	7.2
phenyl-50	283	4.8	12.2	5.1	1.8	6.6

* H_2 diffusion coefficient is given as indication only due to very short time-lag values

Table S4. Solubility coefficient of PIM-1, homo- and copolymers determined at 30°C

	**Solubility coefficient (*10³)**					
	**H_2**	**N_2**	**O_2**	**CO_2**	**CH_4**	**H_2O**
PIM-1	3.9	40	45	760	190	9350
Homopolymer						
methyl-100	4.2	40	45	900	173	15250
ethyl-100	2.4	40	40	800	153	15900
propyl-100	3.6	30	33	675	140	12150
i-propyl-100	-	35	40	725	150	9850
t-butyl-100	3.2	35	35	660	140	8100
cyclohexyl-100	4.4	33	35	670	140	8300
phenyl-100	6.3	30	35	715	125	33500
Copolymer						
methyl-50	5.6	40	47	940	210	21250
ethyl-50	3.3	30	30	625	127	11350
propyl-50	3.7	35	37	705	153	9150
i-propyl-50	3.0	25	33	645	130	19300
t-butyl-50	6.5	40	45	735	160	17250
cyclohexyl-50	4.4	33	40	700	160	8400
phenyl-50	3.9	30	35	630	130	8500

Table S5. Gas transport properties of methyl-100-TT* determined at 30°C

	**H_2**	**N_2**	**O_2**	**CO_2**	**CH_4**	**H_2O**
Permeability (Barrer)	690	105	215	1410	155	32000
Diffusion coefficient (10^7)	127	3.5	6.6	2.2	1.2	1.0
Solubility coefficient (10^3)	5.4	30	33	640	125	34150

*Thermal treatment at 250°C, 2h
